# Retrospective cohort analysis of nitrite and nitrate levels in postmortem biological samples after suspected suicide, 2019-24

**DOI:** 10.1136/bmjph-2025-004215

**Published:** 2026-04-20

**Authors:** Jonathan W Ho, Rohan Hobbs, Nigel Brown, Paul Dargan, Laura J Hikin, Alexander Lawson, Sean Mcgovern, Robert Moore, Paul Smith, Jessica Winfield, Rebecca Wood, Amrita Ahluwalia

**Affiliations:** 1 Barts and the London Faculty of Medicine and Dentistry, Queen Mary University of London, London, UK; 2 Department of Mathematics, King’s College London, London, UK; 3 Toxicology, Clinical Chemistry, Wansbeck General Hospital, Ashington, UK; 4 Clinical Toxicology, Guy's and St Thomas's Foundation Trust, King’s College London, London, UK; 5 Faculty of Life Sciences and Medicine, King’s College London, London, UK; 6 Toxicology Unit, Sheffield Teaching Hospitals Foundation Trust, Northern General Hospital, Sheffield, UK; 7 Biochemistry, Immunology and Toxicology, Queen Elizabeth Hospital, Birmingham, UK; 8 HM Coroner for Coventry, Coventry City Council, Coventry, West Midlands, UK; 9 Department of Toxicology, Royal Sussex County Hospital, University Hospitals Sussex NHS Foundation Trust, Brighton, UK; 10 Forensic Toxicology Service, University Hospitals of Leicester NHS Trust, Leicester, UK

**Keywords:** Adolescent, Emergencies, Humans, Mental Health

## Abstract

**Introduction:**

Globally, reports of suicide by sodium nitrite poisoning have been growing. To determine whether sodium nitrite poisoning in suicide is an issue in the UK, we conducted an analysis of the cohort of cases analysed by the sole provider of nitrite assessment for postmortem sample in the UK.

**Methods:**

Retrospective cohort analysis of biochemical measures for nitrite and nitrate anion in postmortem samples of 201 cases provided by HM coroners of suspected suicide over a period from March 2019 to August 2024. Secondary measures included sample collection and analysis dates, age and sex of deceased, sample number per case, sample types, and coroner regional location.

**Results:**

Of the 201 cases assessed by the laboratory during the inclusive time period, permission to include data was received from HM coroners for 164 of the cases. Suspected suicide by sodium (or potassium) nitrite/nitrate was confirmed in 87% of cases, with measured nitrite and/or nitrate concentrations ~100× normal physiological levels. Sex was known for 98% of cases and 68% were men. The age range was 14–82 years, and most (71%) cases were from Generation Z and Millennial generation. Cases came from across the UK, with the greatest proportion from Greater London, South East England, the Republic of Ireland, and the Midlands.

**Conclusions:**

The data indicate that suicide associated with the ingestion of nitrite or nitrate salts is substantial in the UK, with a disproportionately high prevalence in young men. There is an urgent public health need for policy makers to consider strategies aimed at preventing and mitigating the harms associated with free access to these salts. This could include restrictions to purchase to facilitate prevention and adoption of easy to implement treatment protocols for both prehospital and emergency healthcare staff in suspected suicide by sodium nitrite/nitrate.

**WHAT IS ALREADY KNOWN ON THIS TOPIC**

**WHAT THIS STUDY ADDS**

- This study provides the largest dataset available globally of confirmed suicide by nitrite salt and demonstrates a substantial incidence over the period 2019–24.
- The study highlights young men as users of this method, and importantly the availability of know-how in teenagers.

**HOW THIS STUDY MIGHT AFFECT RESEARCH, PRACTICE OR POLICY**

- The study unequivocally stresses the urgent public health need for policy makers to consider approaches that seek to prevent and mitigate the harms associated with free access to these salts.

## Introduction

**Figure 1:**
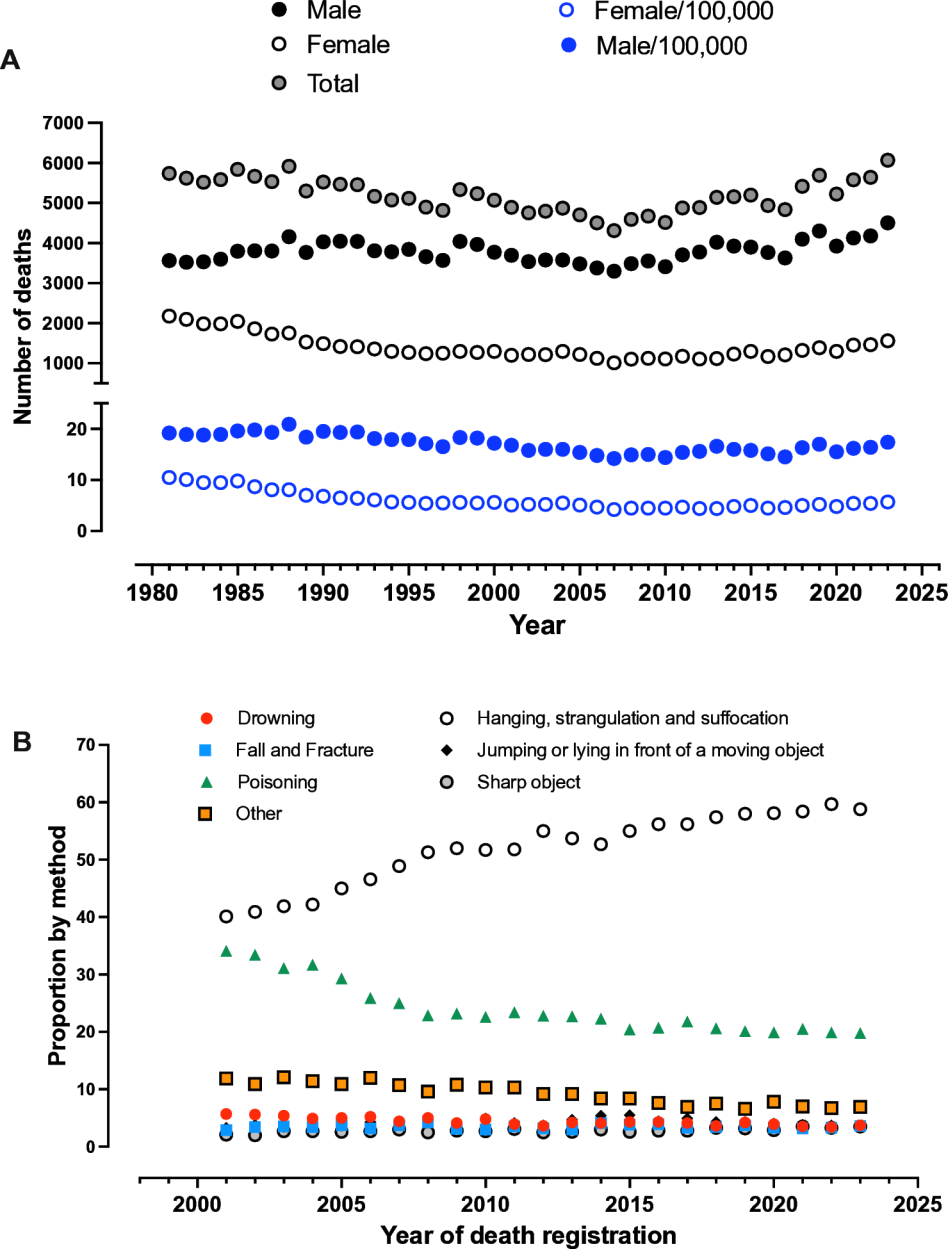
(A) Number of suicide deaths and rate per 100 000 by sex in England and Wales since 1980. (**B**) Proportion of suicides by method in England and Wales, registered 2001–21. Data shown from data tables published alongside the Office for National Statistics’ (ONS) Suicides in England and Wales statistical bulletin and data accessed on 4 April 2025. Death statistics are compiled by the ONS from information supplied when deaths are certified and registered as part of civil registration, a legal requirement.

 Although suicide rates have been decreasing across the UK since the early 1990s, suicide remains a major UK (figure 1) and global public health concern. The Global Burden of Disease study estimated that, in 2021, 746 000 deaths were due to this tragic and preventable cause.^1^ Perhaps one statistic that provides some hope is that the age standardised rate of deaths has been decreasing, with the Global Burden of Disease study indicating a reduction from 14.9 per 100 000 in 2009 to 9 per 100 000 in 2021. Of note, this decrease in rates through to 2021 is also evident in the UK (figure 1A). Although these decreases are encouraging, there is some evidence of a recent uptick in numbers, highlighting the importance of remaining vigilant. The importance of continued focus in this area is exemplified by inclusion of reducing suicide mortality in the United Nations Sustainability Development Goal 3 Targets and Action Plans of the World Health Organization (https://www.who.int/health-topics/suicide#tab=tab_1).

Prevention approaches that seek to reduce suicide rates, both at a personal and population level, require extensive background knowledge of the numerous factors leading to the event.^2 3^ One important component of population level initiatives includes an appreciation of emerging methods of suicide and their availability. Such an appreciation enables the development of policy to not only mitigate the impact of use of such new approaches but also prevention through the introduction of legislation to limit the accessibility of the substance. An example of success in the latter is the introduction of legislation to restrict access to highly hazardous pesticides.^4 5^ These systematic reviews report a reduction in overall suicide mortality in both high and low middle income countries, demonstrating an overall effectiveness in the policy. Figure 1B shows the proportions of the main methods used in suicide in the UK; at first glance, these proportions seem to have remained constant for the past 10 years. However, the data do not reveal potential shifts in the use of any specific approaches within each category. An example of such a change in the past decade is in the poison category. There has been emergence of sodium nitrite (NaNO_2_) ingestion, which results in toxicity due to rising levels of methaemoglobin, leading to cyanosis, cardiovascular collapse and cardiac arrest.

Several recent publications detailing case reports of the use of sodium nitrite in the USA, UK and Canada indicate the availability of information on illicit websites describing how to access and use this salt for the purposes of suicide.^6^,^9^ Indeed, in Canada, an individual has been charged for several offences and first degree murder for their role in promoting and making available sodium nitrite that resulted in the deaths of several individuals across the globe.^7 8^ In the UK, there is a growing awareness of the use of sodium nitrite for suicide, but the scale of the issue is not well appreciated. As the primary laboratory supporting UK coroners in the measurement of the nitrite anion (NO_2_^−^) in postmortem samples,^9^ we are in the unique position of being aware of the potential incidence of the use of sodium nitrite for the purpose of suicide in the UK. In this manuscript, we describe our biochemical assessments analysing levels of nitrite and its oxidised metabolite, nitrate (NO_3_^−^); we also describe the associated demographic information from cases analysed between 2019 and 2024.

## Methods

### Study design

This study is an anonymised retrospective cohort analysis conducted to investigate the characteristics of UK postmortem sample analysis for nitrite and nitrate measurement.

### Sample size, inclusion and exclusion criteria

Since 2019, across the UK, coroners and forensic pathologists have been sending samples from cases where they suspect sodium nitrite may have been used in suicide to the Vascular Pharmacology Laboratory at Queen Mary University of London. Inclusion criteria were all consecutive cases in which nitrite and nitrate levels were biochemically assessed by the laboratory, specifically cases that were received over the period March 2019 to August 2024, with permission from HM coroners for use of the case data. Exclusion criteria were refusals of permission from HM coroners and any cases analysed after August 2024. Detailed information from coroners regarding the basis of their suspicion of sodium nitrite use was not provided.

### Demographic and case data

Postmortem samples analysed in this study were received from HM coroners, toxicologists and police forces within the UK, Ireland and a British overseas territory. Demographic data (biological sex and age) and case data (date of death and date of postmortem) were collected and anonymised. Case data were used to calculate timelines between date of death, date of postmortem and date of nitrite/nitrate analyses. No identifiable information concerning the deceased was used. On receipt at the laboratory, the samples were stored at 4°C until analysis. The median time to analyse the sample after receipt was 5 days (min=0; max=34 days). All samples were discarded within 1 month after analysis. Geographical distribution of cases was classified according to the countries in the UK and Ireland. Distribution in England was further classified by county region. Geographical boundary data were obtained from the Office for National Statistics^10 11^ and Tailte Éireann.^12^

### Sample preparation

Sample types received from the coroners and toxicologists included at least one of the following: blood, vitreous humour, urine, gastric contents or a non-biological liquid found at the scene (table 1). Blood samples were collected in tubes as unpreserved blood or preserved blood, typically with sodium fluoride/potassium oxalate identified as the preservative. Samples remained as received and were stored at 4°C until the day of analysis when a 300–500 µl aliquot of the sample was transferred to a 1.5 mL centrifuge tube. Nitrite and nitrate anion concentrations in aqueous solution were measured as previously described.^13^ Each sample was centrifuged (15 000 rcf, 5°C for 5 min) to separate plasma for analysis in the case of blood samples or potential particulate/cellular content from all other liquid samples. This step was taken to reduce to a minimum any further potential chemistry facilitated by cellular elements and to prevent entry of particulate matter into the chemiluminescence equipment.^14 15^ The colour and consistency of the supernatant and the pellet (if sedimented) of samples were recorded. The supernatant was transferred to a 1.5 mL centrifuge tube and serially diluted using Type1 ultrapure water (Merck Millipore, Darmstadt, Germany). The diluted samples were then stored on wet ice until they were ready for analysis in the nitric oxide (NO) analyser (NOA).

**Table 1:** Summary of samples received in each case

**No of samples supplied per case**	**Total cases**	**No of cases granted permission**
1	150	121
2	36	29
3	11	11
4	3	2
5	0	0
6	0	0
7	1	1
**Sample type**	**Total samples**	**No of samples granted permission**
Blood (unpreserved)	123	102
Blood (preserved)	84	69
Vitreous humour	45	38
Gastric contents	4	4
Urine	12	11
Other non-biological liquid	6	3

* Total No of cases=201; total number of cases=274; No of cases with permission for inclusion in the analysis=164 (82%).

### Nitrite and nitrate measurements

A Sievers 280 NOA (Analytix, Tyne and Wear, UK) was used to measure nitrite and estimate nitrate concentrations in the biological samples. The ozone based chemiluminescent method of nitrite and nitrate analysis was first developed for the analysis of sea water^16^ and then adapted for use in plasma samples.^17 18^ The protocol used is based on the details described in recent articles^19^ and reviews.^20^,^23^ To determine the concentration of nitrite and/or nitrate, samples were subjected to reducing conditions to generate free gaseous NO. This NO was measured using a NOA and is based on the gas phase chemiluminescent reaction between NO and ozone. As 1 mol of NO is generated from 1 mol of nitrite or nitrate, the levels of both anions in a given sample can be accurately estimated. Buffer solutions were bubbled with an inert gas (100% N₂) in a purge vessel. A calibration curve for nitrite or nitrate was constructed by injecting known amounts of sodium nitrite or sodium nitrate. The biological samples were then injected into the purge vessel and concentrations of nitrite or nitrate were calculated.

To determine the nitrite concentration in a sample, the purge vessel contained 0.09 M potassium iodide in glacial acetic acid refluxing under N₂ at room temperature. To measure the total nitrate and nitrite (NO_x_), the sample was added to 0.1 M vanadium (III) chloride in 1 M hydrochloric acid refluxing under N₂ at 95°C to achieve a high conversion efficiency. These conditions result in the reduction of all nitrate and nitrite to NO to give a total NO_x_ value. By subtracting the amount of nitrite measured from the NO_x_ value, an approximate measure of the nitrate concentration was obtained.

### Data analyses

Data were analysed in Microsoft Excel (Microsoft Corporation, Washington, USA) and inbuilt toolboxes in MATLAB R2024b. Cases for the choropleth map were classified geographically using Python (V.3.13) libraries, numpy (V.2.0.2), and geopandas (V.1.1.1). The choropleth map was plotted using the plotly library (V.6.2.0) in Python. All other figures from the case data were plotted using GraphPad Prism 10 (GraphPad Software, Massachusetts, USA). Data are shown as median (IQR) or 95% CI, as appropriate. Typical normal physiological concentrations of nitrite are estimated to be 0.2–0.4 µM. For some analyses, a 100-fold increase above physiological has been taken to indicate a level not typically encountered from the diet.^24 25^ Similarly, a 100-fold increase in nitrate above expected physiological concentrations (20–40 µM) has been assumed to signify an atypical level that is not usually encountered following dietary consumption. As data were not normally distributed, the non-parametric Wilcoxon signed rank test was used to compare the nitrite and nitrate concentrations between paired samples. Statistical significance was set at p<0.05.

## Results

**Figure 2:**
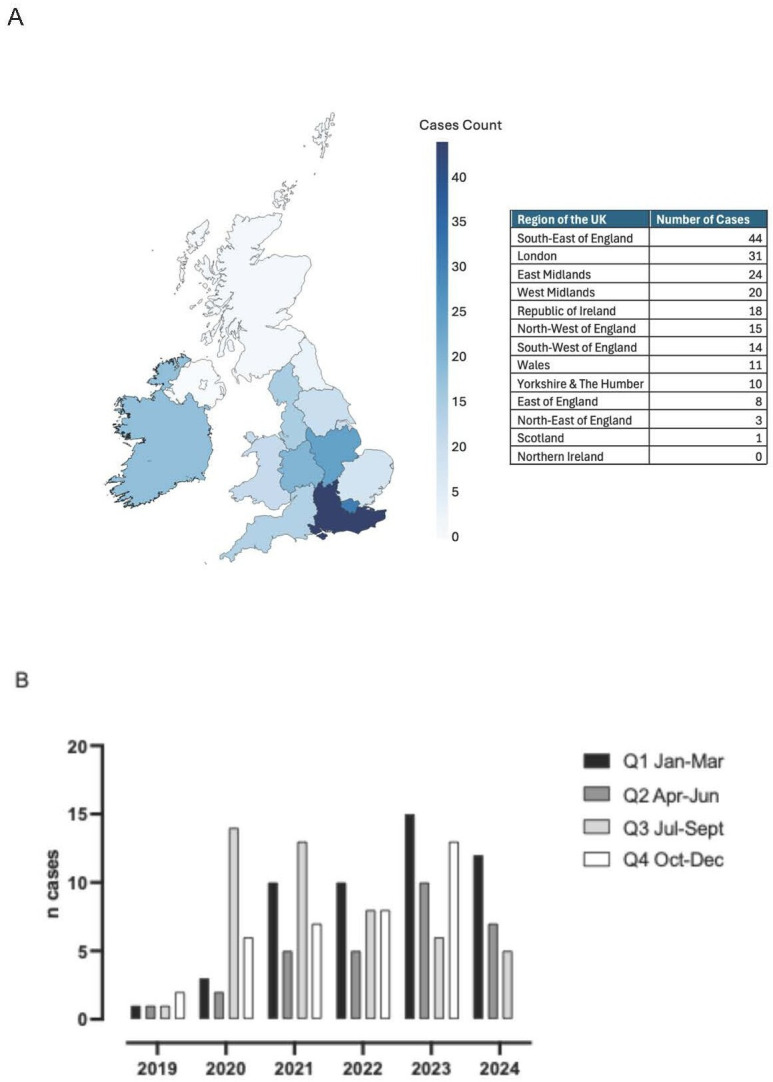
UK regional and number distribution of cases received. (**A**) Choropleth map depicting the regions within the UK where cases were sent from. Cases were received from regions throughout Great Britain and Ireland (table inset). Regional distribution of cases was classified according to region boundary data from the Office for National Statistics (source: Office for National Statistics licensed under the Open Government Licence v.3.0. Contains OS data. Crown copyright and database right 2024) and the Ordnance Survey for Ireland 2025. One case was received from an overseas territory and is not depicted on the map. (**B**) Quarterly number of cases received between March 2019 and August 2024.

A total of 274 samples from 201 cases were received (table 1) where there was a suspicion of unintentional or intentional poisoning between March 2019 and August 2024 from HM coroners, toxicologists and police forces across the UK, Ireland and the Gibraltar British Overseas Territory. Figure 2A is a choropleth map showing the regional density of cases in the UK and Ireland. This indicates that Greater London, South East England, the Republic of Ireland, and the Midlands account for the greatest proportion of cases analysed (figure 2A). Figure 2B indicates the number of cases per quarter over the time period of data collation and demonstrates an annual increase.

The mode sample type was blood. The site of collection of the blood sample was not available to us for most samples and so this detail is not presented here. In several cases, more than one sample type was provided (table 1). Of all the samples, 76% were blood; 59% and 41% of the blood samples were plain (unpreserved) and preserved blood, respectively. The second most received sample type was vitreous humour (16%). Urine and gastric contents constituted 4% and 1% of all the samples received, respectively. Non-biological liquid samples collected at the scene of an incident relevant for a single case made 2% of all samples received (table 1).

**Figure 3:**
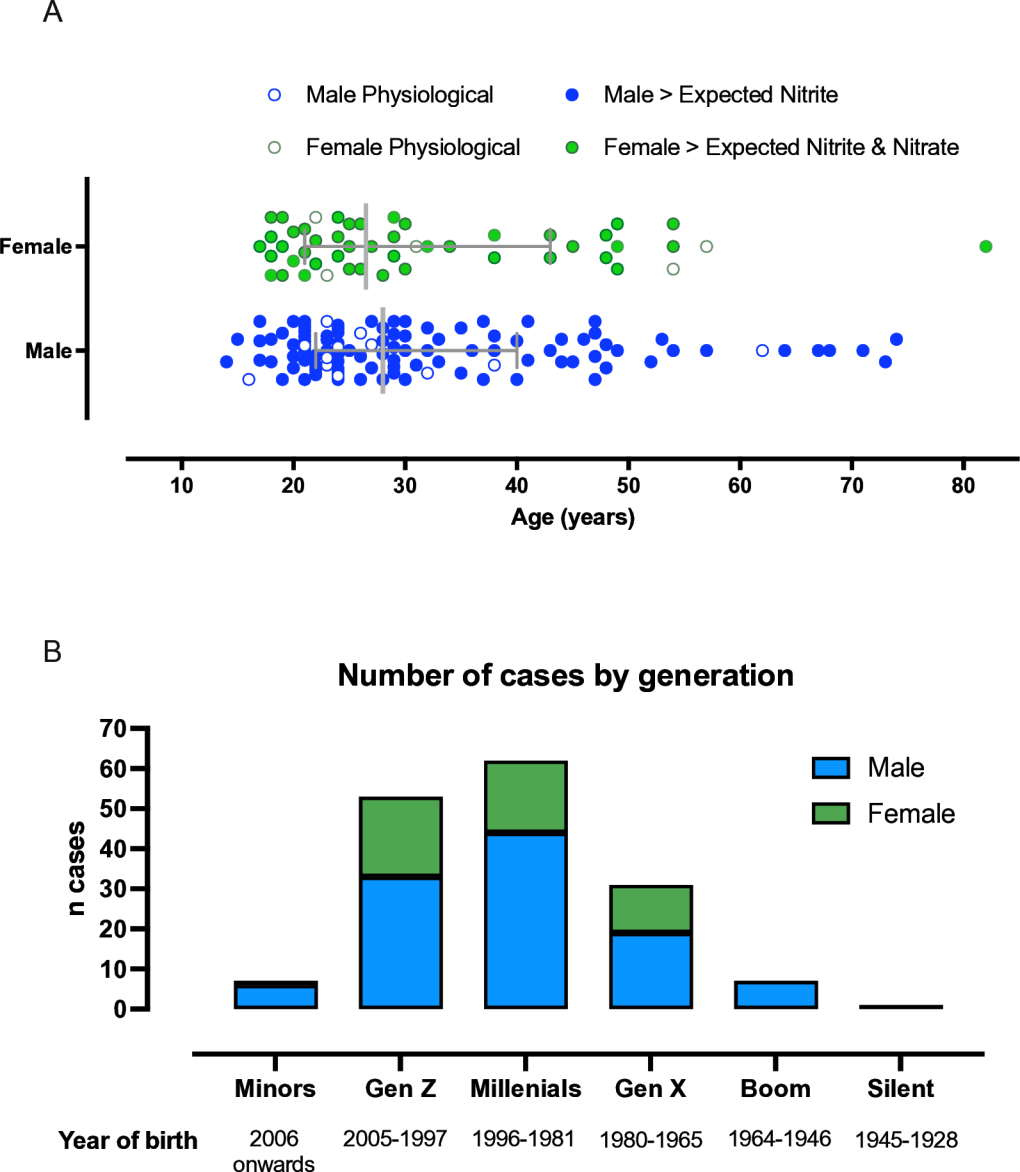
Number of cases by age, generation and sex. (**A**) Grouped scatter graph showing all cases according to sex, with the cases in filled circles shown for those cases where the levels of either nitrite or nitrate measured were deemed to be outside the typical range (ie, unexpected) and the open circles those cases demonstrating levels commensurate with normal physiological levels. Median values with IQRs are shown by the bars. (**B**) Male and female cases grouped by generation. Minors refer to those born after 2006; Generation Z, 1997–2005; Millennial Generation, 1981–96; Generation X, 1965–80; Generation Baby Boom, 1946–64; and Silent Generation, 1928–45.

**Figure 4:**
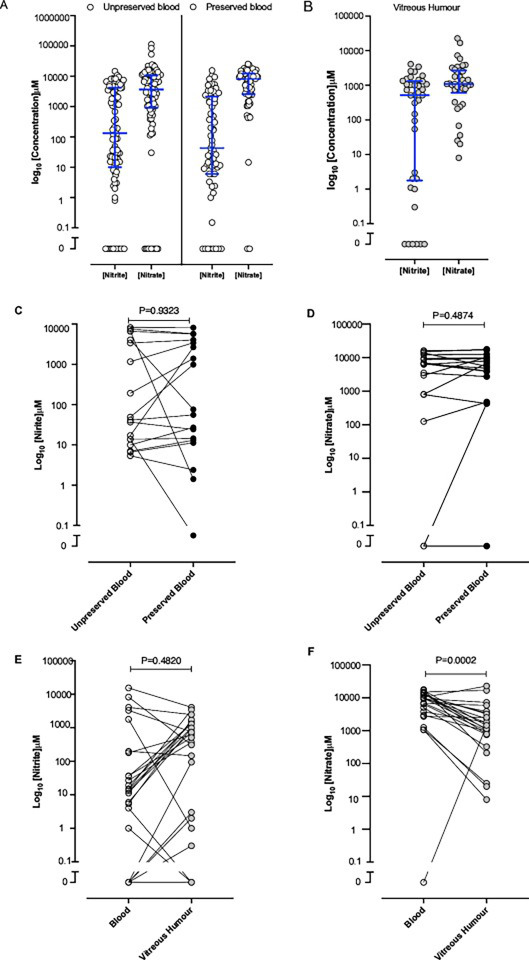
Concentrations of nitrite (NO_2_^−^) and nitrate (NO_3_^−^) measured in postmortem samples**.** Levels of anions in (**A**) plain blood and preserved blood and (**B**) vitreous humour. (**C**) Concentration of nitrite and (D) nitrate in paired unpreserved and preserved blood samples (n=18). (**E**) Concentration of nitrite and (**F**) nitrate in paired blood and vitreous humour samples (n=25). Individual sample measurements are shown with medians (IQRs).

Permission was received from HM coroners to use the case data for 82% of the cases analysed during the specified period (table 1). Data shown in figures 3–4 are from only those cases where formal permission for use was received from the relevant coroners and police forces. Median duration between reported date of death and postmortem was 5 days (IQR 3–5). Median duration between date of postmortem and date of nitrite/nitrate analyses was 54 days (IQR 31–97).

### Sample characteristics

Most of the blood samples were viscous in consistency and brown/burgundy in colour (online supplemental table S1). After centrifugation, it was not possible to generate a clear plasma sample for biochemical analysis from any of the received blood samples. Most vitreous humour samples contained cellular debris; centrifugation resulted in a small white or brown pellet and a clear colourless supernatant. The gastric contents were light green and brown/khaki in colour and turbid. The urine samples were straw coloured and cloudy. Centrifugation of these samples resulted in small white pellets and clear yellow supernatants, which were taken for analyses (online supplemental table S1).

### Demographics

Median age of the cases included in the analysis was 28 years, and this was not significantly different if stratified according to sex. The range of ages for males was 14–74 years and 17–82 years for females (figure 3A and table 2). Due to this wide age range for both sexes, cases were classified into generations defined by the Pew Research Centre^26 27^ and used by the Intergenerational Commission established by the Resolution Foundation: those born after 1997 are often referred to as Generation Z; 1981–96 Millennial Generation; 1965–80 Generation X; 1946–64 Generation Baby Boom; and those born 1928–45 are often referred to as the Silent Generation. Most cases (71 %) were from the younger generations (Generation Z and the Millennial Generation). Concerningly, 4% of cases were minors (aged <18 years at the time of death). The fewest cases were classified in the older generation (Silent Generation=1 %). There were more men than women among the cases (109 men and 52 women). Sex was not supplied in two cases. More than half of the cases in each generation were men, except for the oldest classified generation (Silent), where the only case was a woman (figure 3B).

**Table 2:** Summary demographics of cases received

Demographic characteristics	Number (% or 95% CI)	P value
Sex		0.7553
Men	109 (66.5)	
Women	52 (31.7)	
Median age (years)		
Overall	29.0 (95% CI 26 to 30)	
Men	28.0 (95% CI 22 to 40)	
Women	26.5 (95% CI 24 to 34)	

### Nitrite and nitrate concentrations

Figure 4A shows the wide variation in measured nitrite and nitrate levels in the unpreserved (n=102) and preserved (n=69) blood samples. In unpreserved blood, median concentrations of nitrite and nitrate were 132.2 µM (IQR 10.0–4194.0 µM) and 3647 µM (IQR 911–10 626 µM), respectively. These values were approximately 300–600 times and 90–180 times higher, respectively, than would be expected physiologically. Nitrite and nitrate concentrations in preserved blood samples were 43.0 µM (IQR 6.0–2146.0 µM) and 8144 µM (IQR 2567–12 368 µM), respectively. These concentrations were approximately 100–200 times higher and 200–400 times higher, respectively, than would be expected physiologically.

There were 18 cases with matched plain (unpreserved) and preserved blood samples. For these cases, a within case comparison of nitrite and nitrate concentrations was performed to determine if the use of preservatives impacted the measured concentration of anions (figure 4A). Median nitrite and nitrate concentrations in preserved blood, while slightly higher, were not significantly different than those from plain blood (p>0.05).

Median concentrations of nitrite and nitrate in vitreous humour samples (n=38) were 518.5 µM (IQR 1.8–1316 µM) and 1089.0 µM (IQR 608.3–2697 µM), respectively. These concentrations were approximately 2000 times and 70 times higher, respectively, than would be expected physiologically. Of the cases, 25 were supplied with a matched blood (14 preserved and 11 plain) and vitreous humour sample. Thus a within case comparison of the nitrite and nitrate concentrations between the blood and vitreous humour sample was conducted to determine whether the sample type might influence the concentration of the anions measured (figure 4B). Median concentrations of nitrite and nitrate in the paired blood samples were 14.5 µM (IQR 0.5–189.5 µM) and 9360 µM (IQR 3346–12791 µM), respectively. These values corresponded to approximately 10–15-fold and 300–400-fold higher concentrations, respectively, than would be expected in a fasted healthy individual. In the vitreous humour samples that paired with blood, median concentrations of nitrite and nitrate were 510.4 µM (IQR 1.5–1326 µM) and 1420 µM (IQR 766–3311 µM), respectively. These values represented approximately 100–400-fold and 50–70-fold higher, respectively, above expected physiological levels.

In total, 11 urine samples were provided. Median concentrations of nitrite and nitrate were 65 µM (IQR 1.0–109 µM) and 353 µM (IQR 27–904 µM), respectively. Four gastric contents samples were received. Nitrate was undetectable but median nitrite concentration was 360 mM (95% CI 1.1 M-262 mM). In three cases, an unidentified clear solution was provided. Analyses indicated that although these solutions contained no measureable nitrate, they had extremely high concentrations of nitrite with a median of 0.99 M (range –7.2 M). Of the 164 cases analysed, 21 cases (13 %) did not have elevated nitrite and/or nitrate concentrations.

## Discussion

Although suicide rates have been declining across most of Europe, recent data indicate that rates in the UK (Office for National Statistics data, figure 1) and USA have been increasing.^1 28^ This trend is particularly concerning given that it appears to be driven disproportionately by younger individuals.^3 28^ Intentional poisoning has contributed to these recent increases, and at least in the USA, this rise has been partly attributed to the use (and availability) of sodium nitrite. This trend has emerged alongside freely accessible online information detailing how sodium nitrite can be obtained and used, disseminated both under the guise of providing mental health support and for more explicitly harmful purposes.^6^ In the UK, the scale of the use of sodium nitrite has yet to be fully clarified due to an absence of information. Here we showed that the number of cases sent to our laboratory by forensic services and coroners, where use of sodium nitrite was suspected, has risen substantially since 2019 when we began receiving samples for assessment. Importantly, 87% of these cases showed extremely high concentrations of nitrite and/or nitrate anions, providing strong biochemical support for suspected ingestion. Collectively, these findings establish unequivocally that use of sodium nitrite in the UK as a method of suicide is both substantial and concerning.

We showed that conventional blood sample collection (ie, in the absence of preservatives) provided samples of sufficient quality to measure nitrite and nitrate concentrations and allowed us to infer whether these concentrations could have been due to external ingestion (eg, sodium and potassium salts). Importantly, collection of blood into a preservative had minimal impact on conclusions derived from the analysis. As noted, circulating concentration of nitrite in the blood of a healthy individual, who has fasted for approximately 8 hours before collection, is about 0.1–0.4 µM, whereas circulating nitrate concentrations are generally 20–40 µM.^29^,^32^ This nitrite is derived predominantly (~80–90%) from the conversion of dietary nitrate from within consumed vegetables, particularly green leafy vegetables and beetroot,^24 33^ representing the major dietary source. The oxidation of endogenously generated NO accounts for 5–10% of circulating nitrite concentrations. Very small proportions of <5% of nitrite arise directly from dietary sources (mainly from processed meat).^25^ Levels of nitrite in blood are further constrained by its high reactivity with oxyhaemoglobin, which converts nitrite to generate nitrate and methaemoglobin.^14 15^ This reaction may also occur following blood collection and during sample storage before analysis. A key limitation of the findings is that the measured values may not accurately reflect the true *in vivo* concentrations before death. Evidence of haemolysis was evident in many blood samples, indicated by the cellular debris pellet after centrifugation and the pink–red discolouration of the plasma, indicative of free haemoglobin released during red blood cell lysis. This free haemoglobin is then available for interactions with nitrite. Such a reaction would increase the levels of nitrate measured, while decreasing the levels of nitrite and lead to substantial variability in levels measured, dependent on sample quality, as has been acknowledged recently elsewhere.^34^

A small proportion of circulating nitrate is typically derived from the oxidation of endogenously generated NO (as with nitrite), with most coming from the diet, typically approximately 75%.^24 35^ Importantly, increasing circulating nitrate levels 10-fold through dietary nitrate intake causes an approximate doubling of circulating nitrite concentrations through the activity of the enterosalivary circuit.^24^ The ratio of circulating concentrations of nitrate to nitrite is typically 100:1. In most of the cases analysed, the ratio was 10–100-fold higher. On some occasions, the ratio was substantially below 1. Such results strongly indicate ingestion of nitrite particularly in the latter when the ratio is <1. For the former, there may be two explanations: potential ingestion of nitrate or, more likely, that nitrate levels have been derived from the oxidation of nitrite. Most importantly, substantial distortions of this ratio indicate sources of the nitrite or nitrate anions other than conventional dietary intake shortly before death.

Absence of elevated levels of either anion in 13% of cases may be explained by several possibilities. Firstly, the initial suspicion of sodium nitrite ingestion may have been incorrect, an explanation that is most plausible in those instances where neither nitrite nor nitrate showed any elevation. Secondly, in a subset of cases where nitrite concentrations appeared to fall within the expected physiological range, nitrate concentrations were modestly elevated but did not exceed the 100-fold threshold. A plausible explanation is that ingested nitrite underwent near complete oxidation to nitrate before sampling, thereby reducing detectable nitrite while still producing a measurable, although not extreme, increase in circulating nitrate levels. Experimental evidence supports this possibility: raising circulating nitrite concentrations in healthy volunteers to approximately 3–4 µM by continuous sodium nitrite infusion has been shown to reduce blood pressure by >15 mm Hg and increase methaemoglobin levels to 3–5%^36^. Cyanosis typically becomes clinically apparent at levels of 10% methaemoglobin. If all of the infused nitrite were subsequently oxidised by oxyhaemoglobin, circulating nitrate concentrations would be expected to rise proportionately. Thus seemingly modest elevations in nitrate may in fact reflect exposure to nitrite at levels capable of causing substantial physiological toxicity. This interpretation raises the possibility that the 13% of cases classified as not supporting sodium nitrite ingestion may represent an underestimate.

Comparing preserved with unpreserved blood measurements indicated little difference in both anion levels. Interestingly, however, in the 25 cases where both a blood and vitreous humour sample were provided, significant differences between the blood and vitreous concentrations were evident. There are several estimates of levels of both anions in vitreous humour in the literature. Two studies comparing healthy individuals with patients with diabetes identified levels of nitrite ranging from 0.3 to 2.0 µM^37^ and nitrate of 15 μM,^38^ levels not dissimilar to those measured in blood. The absence of haemoglobin in vitreous samples, and thus less possibility for post-collection chemistry compared with blood, we suggest provides a sample type that is less prone to post-processing chemistry and thus artefacts introduced during storage and handling. However, vitreous humour does contain proteins, approximately 1.2 mg/ml,^39^ of which a substantial proportion is collagen, and an estimated 450 different proteins.^40^ It is therefore conceivable that nitrite present in vitreous humour samples may react chemically with protein residues, such as tyrosine or thiol, before the biochemical analyses. Such reactions typically require favourable conditions, including oxidative stress environments and acidosis. At least for the latter, the vitreous samples tested in the dataset showed neutral pH, making these reactions less likely to occur in practice.

Comparing matched blood with vitreous humour samples revealed an important advantage of using vitreous humour samples over blood i.e., the relative lack of haemoglobin in vitreous humour. Consistent with this, vitreous samples tended to show higher concentrations of nitrite and comparatively lower nitrate levels than paired blood samples. This pattern may be explained by post-processing oxidation of nitrite to nitrate in blood during storage, a process far less likely to occur in haemoglobin-free vitreous humour. These results suggest that optimum assessment of potential nitrite poisoning is likely achieved using vitreous humour samples. In contrast, urine samples did not often show elevated nitrite or nitrate levels, even in cases where blood levels of nitrite and/or nitrate were high. A potential explanation for this difference is that the lethal consequences of substantial external ingestion of nitrite and/or nitrate is so rapid that there is insufficient time for renal clearance of either anion into urine. A possibility consistent with the rapid lethality of sodium nitrite ingestion. Supporting this, reports show that sodium nitrite has high oral bioavailability, with absorption from the gastrointestinal tract estimated to be 90–98% and occurring within minutes following ingestion .^41^ High levels of circulating nitrite concentrations are likely to cause profound decreases in blood pressure at levels potentially leading to circulatory collapse and cardiac arrest. In addition, the high levels of nitrite measured will have inevitably been accomapnied by clinically dangerous, indeed catastrophic, rises in methaemoglobin levels^36 42^ leading to cyanosis. Both phenomena, if occurred, will likely have contributed to death.

The age range of the deceased was wide, with the youngest aged 14 years and the oldest 82 years. However, the majority of the cohort tended to be younger individuals, with 38% of all cases born after 1997 (Millennial Generation) followed by 33% born in 1981–96 (ie, Generation Z). The age range suggests that access to substantial quantities of the salts of these anions is not age limited but perhaps younger generations are more aware of this poison approach. Most recently, the news media has highlighted individuals in Canada^8^ and Ukraine^7^ selling sodium nitrite online connected with websites promoting suicide. Our data provide strong support for the suggestion that the improved digital literacy of younger people enables access to illicit online material promoting suicide practices and lends further support for calls for tighter legislation to prevent availability of such information in online forums.

Previous suggestions that the incidence of nitrite/nitrate assisted suicide is rare in the UK are not supported by our findings. The choropleth map demonstrated that cases seemed to be concentrated in specific regions, with most cases arising from the South East of England, the Midlands and Southern Ireland. However, interpretation of these geographical patterns requires caution. Awareness of our laboratory’s analytical support in analysis of nitrite and nitrate has spread largely through professional networks and by word of mouth. Thus regional coroners' uptake of testing may reflect varying levels of awareness rather than true incidence. In addition, many of the forensic services that send cases for analysis to our laboratory assess cases originating outside of the geographical region in which those services are based. A clearer understanding of the true prevalence and regional distribution will likely only be forthcoming if assessment of nitrite and nitrate levels become routine postmortem toxicological practice in cases of suspected suicide.

Importantly, while the cases presented here relate to postmortem analysis, there are reports in the literature describing individuals who have sought emergency help following ingestion.^43^,^50^ In those cases where patients survived, this was attributable to treatment with vasopressors and methylthioninium chloride (commonly known as methylene blue). Reduction of methaemoglobin to haemoglobin is essential to restore oxygen binding and transport. Under normal physiological conditions, methaemoglobin is reduced via the activity of cytochrome b5 reductase and nicotinamide adenine dinucleotide phosphate MetHb reductase. However, following exposure to the massive doses evidenced in our analyses, this endogenous pathway becomes saturated, resulting in a rapid rise in methaemoglobin levels. Methylthioninium chloride acts as a cofactor for cytochrome b5 reductase, thereby restoring its reductive capacity.^46 51^ The provision of methylthioninium chloride kits in ambulances in the advent of potential rises in intentional poisoning through this method has been mooted as a simple and cost effective timely method to prevent the devastating consequences of ingestion.^46^ Indeed, recent evidence from a small retrospective pilot trial, during a planned evaluation period, of its availability in ambulances where sodium nitrite use was suspected, found that four out of nine patients survived to arrival at hospital and three survived long term.^52^ It is worth noting that methaemoglobin levels in a living person are relatively easily estimated using the non-invasive method of co-oximetry. However, this measurement is not generally used to identify cause of death (although may be used to confirm other analysis) due to the unreliability of the method postmortem.^53^

### Limitations of this study

There are some important limitations that should be considered. Firstly, the summary analysis was derived solely from cases submitted by coroners, pathologists, or police forces to the laboratory, where there was some suspicion that sodium nitrite may have been ingested. This results in a bias, overt physical evidence as well as awareness of the forensic team of our services have inevitably influenced the high positive rate encountered. Inclusion of all consecutive cases in this analysis limits any further bias in selection. To preserve anonymity, no case specific contextual information was shared with the laboratory. Anecdotally, suspicions often arise from evidence of online activity or the presence of an unidentified white powder or related packaging at the scene. However, because nitrite and nitrate analysis is not routinely mandated for all suspected suicides, the true incidence of sodium nitrite use in the UK remains uncertain. It is therefore likely that the cases included here represent a substantial underestimate of the actual incidence. Secondly, the interval between death and sample receipt varied considerably, introducing the possibility that delays may have affected the accuracy of the biochemical measurements.

## Conclusions

The presented data suggest that analysis of postmortem samples for nitrite/nitrate levels is a valuable method for confirming death associated with nitrite ingestion. We showed that vitreous humour tends to provide the optimum sample for analysis postmortem. Our findings also demonstrated that suicide related to ingestion of nitrite salts was frequently observed in suspected cases in the UK, particularly in those involving young men. These observations highlight an urgent public health need for policy makers to consider implementing measures to prevent and mitigate the harms associated with unrestricted access to these salts. Potential measures include restrictions to availability and adoption of treatment protocols for both prehospital and emergency healthcare providers in cases of severe methaemoglobinaemia related to ingestion of these salts.

## Supplementary material

10.1136/bmjph-2025-004215 online supplemental file 1

## Data Availability

Data are available upon reasonable request.
