# Supplementary material for: Retrospective cohort analysis of nitrite and nitrate levels in postmortem biological samples after suspected suicide, 2019-24
Source: BMJ Public Health. 2026 Apr 20;4(2):e004215. doi: 10.1136/bmjph-2025-004215 (PMC13140958; doi:10.1136/bmjph-2025-004215)
Supplement: online supplemental file 1 [file bmjph-4-2-s001.docx]

**Supplementary Data**

**A retrospective cohort analysis of nitrite and nitrate levels in postmortem biological samples following suspected suicide between 2019-2024: evidence highlighting a need for urgent action in the UK**

Jonathan W. Ho^1^, Rohan Hobbs^2^, Nigel Brown^3^, Paul Dargan^4^, Laura J. Hikin^5^, Alexander J. Lawson^6^, Sean McGovern^7^, Robert Moore^8^, Paul Smith^9^, Jessica Winfield^6^, Rebecca Wood^8^, Amrita Ahluwalia^1^

^1^Barts & The London Faculty of Medicine & Dentistry, Queen Mary University of London, Charterhouse Square, London EC1M 6BQ, UK

^2^ Department of Mathematics, King’s College London, London, UK

^3^Toxicology, Clinical Chemistry, Wansbeck General Hospital, Ashlington, Northumberland, UK

^4^ Clinical Toxicology, Guy’s and St Thomas’ NHS Foundation Trust, London, UK

Faculty of Life Sciences and Medicine, King’s College London, London, UK

^5^Toxicology Unit, Sheffield Teaching Hospitals Foundation Trust, Northern General Hospital, Herries Road, Sheffield, UK

^6^ Biochemistry, Immunology and Toxicology, Queen Elizabeth Hospital, Birmingham, UK

^7^HM Coroner for Coventry, Coventry City Council, West Midlands, UK

^8^ Department of Toxicology, University Hospitals Sussex NHS Foundation Trust, Royal Sussex County Hospital, Brighton, UK

^9^ Forensic Toxicology Service, University Hospitals of Leicester NHS Trust, Leicester Royal Infirmary, Infirmary Square, Leicester, Leicestershire, UK

Author for correspondence:

Prof Amrita Ahluwalia

Barts & The London Faculty of Medicine & Dentistry,

Queen Mary University of London,

Charterhouse Square,

London EC1M 6BQ, UK

Supplement Table S1. Physical description and appearance of the biological samples received for all cases

| **All blood samples** | |
| --- | --- |
| **Colour & Consistency** | **Number** |
| Red/claret & Thin | 27 |
| Brown/burgundy & Thin | 25 |
| Red/claret & Viscous | 46 |
| Brown/burgundy & Viscous | 69 |
| No colour documented; Thin | 0 |
| No colour documented; Viscous | 25 |
| Red/claret; consistency not documented | 5 |
| Brown/burgundy; consistency not documented | 2 |
| No colour or consistency documented | 8 |
|  |  |
| **Vitreous humour samples** | |
| **Description** |  |
| Contained cellular debris | 28 |
| Clear colourless and contained no cellular debris | 3 |
| Brown/red liquid with or without cellular debris | 2 |
| No description documented | 12 |
|  |  |
| **Gastric contents** | |
| **Description** |  |
| Light green | 1 |
| Brown/khaki and turbid | 1 |
| No description documented | 2 |
|  |  |
| **Urine** | |
| **Description** |  |
| Straw coloured & cloudy | 8 |
| No description documented | 4 |
|  |  |
